# Impact of Structural Dimensionality on the Optoelectronic Behavior of Lead–Halide Perovskites

**DOI:** 10.3390/ma19101990

**Published:** 2026-05-11

**Authors:** Hamida Gouadria, Jesús Álvarez, María José Capitán

**Affiliations:** 1 Departamento de Física de la Materia Condensada, Universidad Autónoma de Madrid, 28049 Madrid, Spain; hamida.goidria@imdea.org (H.G.); jesus.alvarez@uam.es (J.Á.); 2 Instituto Madrileño de Estudios Avanzados en Nanociencia, c/Faraday, 9, 28049 Madrid, Spain; 3 Física de Sistemas Crecidos Con Baja Dimensionalidad, Universidad Autónoma de Madrid, Associated Unit to IEM-CSIC, DP, 28006 Madrid, Spain; 4 Instituto de Ciencia de Materiales “Nicolás Cabrera”, Universidad Autónoma de Madrid, 28049 Madrid, Spain; 5 Condensed Matter Physics Center (IFIMAC), Universidad Autónoma de Madrid, 28049 Madrid, Spain; 6 Instituto de Estructura de la Materia IEM-CSIC, c/Serrano 121, 28006 Madrid, Spain

**Keywords:** lead–halide perovskite, photocurrent, electron confinement

## Abstract

This study investigates how structural dimensionality affects the optoelectronic behavior of organic lead–halide hybrid perovskites. Using the chiral cation R-1-phenylethylammonium (PEA), which is known to be able to form both one-dimensional (1D) and two-dimensional (2D) lead–iodide frameworks, we synthesize 1D $(\mathrm{PEA}){\mathrm{PbI}}_{3}$ and 2D ${\left(\mathrm{PEA}\right)}_{2}{\mathrm{PbI}}_{4}$ compounds through tailored crystallization and deposition routes. X ray diffraction confirms structural purity, while ultraviolet photoelectron spectroscopy (UPS) provides insight into the electronic structure and photoresponse. Both materials exhibit a surface photo-voltage (SPV) under visible illumination, reaching a maximum work function shift of 1.5 eV for the 2D phase and 0.4 eV for the 1D phase in the thin-film samples. These results suggest that the 1D phase exhibits a reduced tendency for iodide-vacancy formation, which may result in a more stable response under visible illumination, accompanied by faster relaxation dynamics and more anisotropic charge transport. Overall, our findings highlight the central role of electronic confinement in shaping photoinduced processes in hybrid perovskites and support the consideration of structural dimensionality as a key design parameter for the design of next-generation optoelectronic materials.

## 1. Introduction

Hybrid organic–inorganic metal halide perovskites (HOIP) have emerged as a highly versatile class of optoelectronic materials. As a result, the number of studies on hybrid organic–inorganic metal halide perovskites has grown exponentially since the publication in 2012 of two breakthrough papers demonstrating their efficiency for solid-state devices [1,2]. Since these initial reports, perovskite solar cells (PSCs) have rapidly achieved certified power conversion efficiencies (PCEs) that exceed 25% [3], while benefiting from low-temperature, solution-based processing routes [4,5]. Their combination of strong absorption, defect tolerance, and exceptional charge-carrier diffusion has fueled intense interest in developing perovskites for next-generation optoelectronic technologies [6,7]. As a result, PSCs are now considered a leading candidate for low-cost, high performance solar energy deployment worldwide [8,9,10].

In order to achieve further advances, it is important to determine how different factors affect their photoelectronic properties and long-term operational stability for perovskite-based devices [11,12,13]. Under continuous illumination, these materials exhibit slow relaxation dynamics arising from their soft ionic lattice, high density of defects, and mixed ionic–electronic transport. Trap states promote charge trapping/detrapping processes, while the migration of mobile ions modifies internal electric fields, leading to hysteresis and reduced charge-extraction efficiency [14,15,16]. These coupled processes strongly influence electron–hole recombination kinetics and ultimately limit device stability and reliability [17,18,19]. Elucidating the interplay between charge transport, recombination, and ionic motion is therefore essential to tailor the design of these materials. This enables the possibility of modulating these properties, allowing an “*ad hoc*” design of perovskites according to the desired final properties [16].

Hybrid lead–halide perovskites continue to attract broad interest due to their slow charge-carrier dynamics, strong absorption coefficients, and remarkably long carrier lifetimes [20,21,22,23,24]. Moreover, their electronic properties can be systematically tailored via chemical modification of both organic and inorganic sublattices, enabling rational design of materials with application specific optoelectronic properties [16]. However, there are still open challenges associated with perovskite materials, in particular, those related to long-term stability, ion migration, defects, and reliability. In this direction, the slow relaxation dynamics, defects, mixed ionic–electronic transport, hysteresis, and reduced device stability remain open issues [25]. The dimensional engineering of the Pb–halide network provides a direct and powerful handle to modulate confinement, carrier-transport pathways, and recombination efficiencies [26,27,28,29,30]. However, the mechanistic consequences of transitioning between 1D and 2D PbI frameworks remain insufficiently resolved, despite their potential to significantly influence light absorption, carrier extraction, and photostability in optoelectronic devices [31,32].

Controlling the dimensionality of the inorganic framework provides a powerful means to tune the optoelectronic response of hybrid perovskites. Depending on the organic cation and synthesis conditions, Pb–halide networks can form one-dimensional (1D) chains, two-dimensional (2D) layered structures, or three-dimensional (3D) frameworks. Dimensional reduction enhances quantum and dielectric confinement, modifying exciton binding energies, band dispersion, and charge-transport anisotropy [33]. In 2D perovskites, carriers are confined within Pb–halide layers separated by organic barriers, yielding strong excitonic features and restricted interlayer transport [33,34]. In 1D architectures, confinement along linear chains produces highly anisotropic diffusion and distinct recombination pathways [35]. These structure-dependent effects offer an attractive strategy for controlling carrier migration and recombination.

In this work, we investigate a series of hybrid lead–halide perovskites capable of self-assembling into 1D or 2D PbI frameworks to evaluate how confinement dimensionality dictates charge-carrier dynamics. By isolating the structural contributions of the inorganic scaffold, we study how dimensionality influences light absorption, carrier mobility, diffusion lengths, and recombination lifetimes. Understanding these relationships is essential for establishing design principles that connect perovskite composition, inorganic dimensionality, and photoactive behavior.

Here, we establish a structure–property framework that correlates confinement dimensionality with charge-carrier transport and recombination processes in PbI-based perovskites. Our results suggest how controlled dimensional reduction of the inorganic component can be leveraged to access customized optical and electronic responses. This work provides a proof of concept for the deliberate design of perovskite materials with tailored photoactive properties and highlights a route toward next-generation, stability-optimized photovoltaic systems.

## 2. Materials and Methods

### 2.1. Synthesis

**Figure 1:**
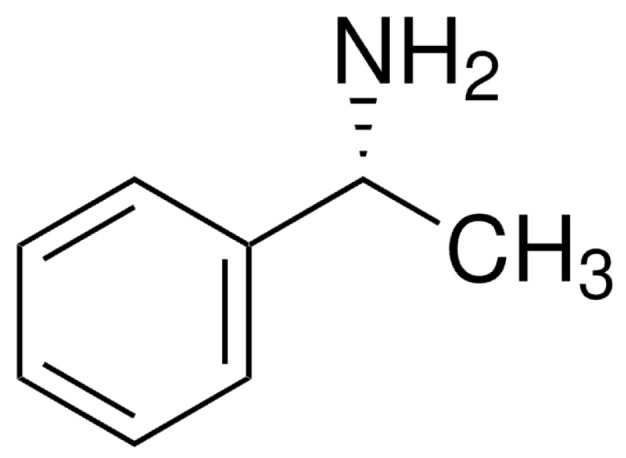
Organic R-1-PhenylEthylAmine (R-PEA) enantiomer molecule used in this study.

In this work, we will focus on the organic R-1-PhenylEthylAmine (R-PEA) enantiomer (see Figure 1) because we did not find any structural or electronic differences compared to its enantiomer using the techniques used in this work. Thus, we refer to the enantiomer R-PhenylEthylAmine as PEA from now on. In this study, we obtained Lead–Iodide perovskites containing this PEA organic chain using different synthesis conditions to obtain 2D and 1D phases of this system in different sample forms (powder, single crystals, and thin films). The main objective is to have a comparative study of how the electronic behavior of the same system changes when its structure changes from wires (1D phase) to planes (2D phase) without changing the chemical components of the system. We hypothesize that this dimensional change in the inorganic part will significantly affect the conduction properties, even modifying the macroscopic and quantum characteristics of a free-electron gas when moving from a 2D to a 1D system. To demonstrate the aforementioned relationship, it is necessary to identify compounds that share the same chemical composition but differ in their structural dimensionality. In this context, we found that the pair (R-${\mathrm{PEA})}_{2}{\mathrm{PbI}}_{4}$ (two-dimensional structure) and (R-$\mathrm{PEA}){\mathrm{PbI}}_{3}$ (one-dimensional structure) are excellent candidates, as both contain the same organic molecule and inorganic elements while preserving a similar composition.

To prepare the R-PEA Lead–Iodide samples (${\left(PEA\right)}_{\left(2-x\right)}{\mathrm{PbI}}_{\left(4-x\right)}$, where x takes a value of 0 or 1 depending on the crystallization temperature treatment) we used the slow evaporation method. This technique consists of dissolving 200 mg of PbO (Sigma-Aldrich powder of 99.9% purity, St. Louis, MO, USA) in 6 mL of HI (Sigma-Aldrich 57% in aqueous solution 99.99%), followed by the addition of 200 μL added drop by drop R-1-phenylethylammine (Sigma-Aldrich, liquid 98%). The solution obtained was heated to ${100\;}^{{}^{\circ}}$C for 3 h. The 2D-${\left(PEA\right)}_{2}PbI_{4}$ phase is stable at room temperature, and it was easily obtained. However, a stable one-dimensional phase, identified as $(PEA){\mathrm{PbI}}_{3}$, requires treatment at high crystallization temperatures. Thus, two different heating treatments were used: to obtain a perovskite stable at room temperature, the solution was cooled directly to room temperature; whereas to obtain the compound stable at a higher temperature, the dissolution was cooled to ${85\;}^{{}^{\circ}}$C for 4 days and then to ${60\;}^{{}^{\circ}}$C for 1 day, without agitation. Finally, the solids were dried under vacuum for 24 h, obtaining the desired samples ${\left(PEA\right)}_{\left(2-x\right)}{\mathrm{PbI}}_{\left(4-x\right)}$ [36]. The powders were obtained by milling in an agate mortar.

After synthesizing the material, two techniques were used for the preparation of perovskite thin films.

1. “Spin coating” technique: this method consisted of dissolving R-1-phenylethylammonium lead–iodide in acetone at room temperature until a saturated solution was obtained. In this way, the solution concentration is given by the Solubility Product Constant of the perovskite, ensuring reproducibility across all experiments. Then, 200 μL of the prepared solution were applied dropwise onto a well-cleaned glass substrate coated with indium tin oxide (ITO) and a layer of gold. The substrate, previously cleaned in an ultrasonic bath with acetone/ethanol cycles and dried under $N_{2}$ gas flow, was placed in the center of the spin coater, which rotated at 400 rpm for 30 s. The resulting thin film was then left to dry at room temperature for 24 h.
2. Deposit on heated substrate: on the other hand, the solution prepared in the same way was deposited on a glass substrate that was also well cleaned and coated with ITO and a layer of gold, but in this case, the substrate was placed on a heater until a temperature of about ${80\;}^{{}^{\circ}}$C, facilitating the formation of the thin film. Finally, the thin film was also allowed to dry at room temperature.

### 2.2. Structure and Electronic Characterization

Powder X-ray diffraction data were collected using a Siemens D5000 diffractometer equipped with a Cu anode (Siemens AG, Munich, Germany). The diffractometer was provided with a ϕ-axis rotation, with a speed of 4 Hz, for the powder samples measurements. The detector arm was equipped with a graphite monochromator to remove fluorescence and $K_{\beta }$, and an automatic attenuator device [37]. The sample powders were milled in an agate mortar and deposited in a zero-background Si sample holder. The diffracted signal was recorded using a NaI(Tl) scintillator from Oxford Instruments. The amplified detector signal was processed using a tunable differential discriminator with a reconfigurable energy window in order to select useful events. The detector energy window was selected with the aid of a multi-channel analyzer. With this setup, the diffracted data were collected using both Cu $K_{\alpha_{1,2}}$ anode lines. The Cu $K_{\alpha_{2}}$ line was removed after data collection using a mathematical procedure based on a fit with penalized splines [38]. The obtained diffraction pattern was indexed using the N-TREOR9 software [39] integrated in the EXPO2014 package [40].

The structure refinement consisted of two steps: first, the position of the Pb and I atoms that form the octahedra was determined by the direct methods integrated in EXPO2014. Secondly, with the octahedra position determined, the organic molecules were introduced into the unit cell, and their positioning was refined using the simulated annealing procedure implemented in EXPO2014. A final step of global refinement was performed. Although we have made a complete refinement of the structures, we have not recorded them in the Cambridge Crystallographic Data Base as a new entry because we found that they essentially agree with the crystal data refs. 607736 and 607737 for the 1D-phase and 607740 and 607741 for the 2D-phase given by Billing et al. [36].

The electron spectroscopy experiments reported in this paper have all been carried out under ultra-high vacuum (UHV) conditions. The sample temperature is monitored by means of a type K thermocouple in direct contact with the sample holder. The experimental chamber has a base pressure of $2\times 10^{-10}$ mbar and is equipped with a He discharge lamp that provides He-I (hν = 21.2 eV) and He-II (hν = 40.8 eV) photons for ultraviolet photoemission spectroscopy (UPS). A hemispherical energy analyzer (LEYBOLD LHS10) was used. The pass energy of the analyzer was set to 5 eV for UPS measurements, providing a resolution of 0.1 eV.

The energies of the UPS spectra were also referenced to the Fermi edge of the clean Ag(100) crystal. The single-crystal Ag(100) was then prepared in situ by ${\mathrm{Ar}}^{+}$ ion sputtering cycles with a current density of 2 $\mu {A/\mathrm{cm}}^{2}$ and annealing at 900 K, until negligible contamination was detected on the surface and a sharp LEED pattern was observed.

For the light on–off transient experiments, a 5W “white” LED source with an equivalent blackbody color temperature of T=4000 K was used for sample illumination. The detailed spectral composition of the radiation has been described elsewhere [41]. The light enters the UHV chamber through a standard UHV viewport with flat transmission in the visible wavelength range. The source is located at a distance of 30 cm from the sample, with an incidence angle of $35^{{}^{\circ}}$ relative to the surface plane. The light beam divergence is $38^{{}^{\circ}}$. Under these conditions, the power density incident on the sample is below $4\;\mathrm{mW}{\mathrm{cm}}^{-2}$.

In addition, a three-component RGB laser with a total power of 3 W (1 W per component) was used. Each component is centered at $\lambda_{\mathrm{red}}=635\;\mathrm{nm}$, $\lambda_{\mathrm{green}}=520\;\mathrm{nm}$, and $\lambda_{\mathrm{blue}}=450\;\mathrm{nm}$. The spectral bandwidth of each component is typically 6 nm. The laser beam was optically expanded at the sample position to an area of $91\;{\mathrm{cm}}^{2}$ to ensure homogeneous illumination of the sample, resulting in a power density of $9\;\mathrm{mW}\;{\mathrm{cm}}^{-2}$.

All measurement techniques used in the present work (X-ray diffraction and photoelectron spectroscopies) are event-counting methods (photons in the case of X-ray diffraction and electrons in the case of photoelectron spectroscopy) and therefore follow Poisson statistics, which evolve toward a Gaussian distribution at high counting rates. In such cases, the uncertainty associated with the measured variables corresponds to the square root of the number of detected events [42]. As part of the instrument calibration procedure, we verified that the standard deviation of the measured data matches the expected Poisson–Gaussian square-root behavior.

## 3. Results

### 3.1. Structural Results

This section presents the structure of the hybrid perovskite samples as determined by X-ray diffraction techniques (XRD). Once the samples were prepared, the diffraction patterns were analyzed to identify the crystalline phases present and to evaluate the structural characteristics under the different experimental conditions used. Here we show the results separated for powder and thin-film samples.

#### 3.1.1. Powder Samples Structure

**Figure 2:**
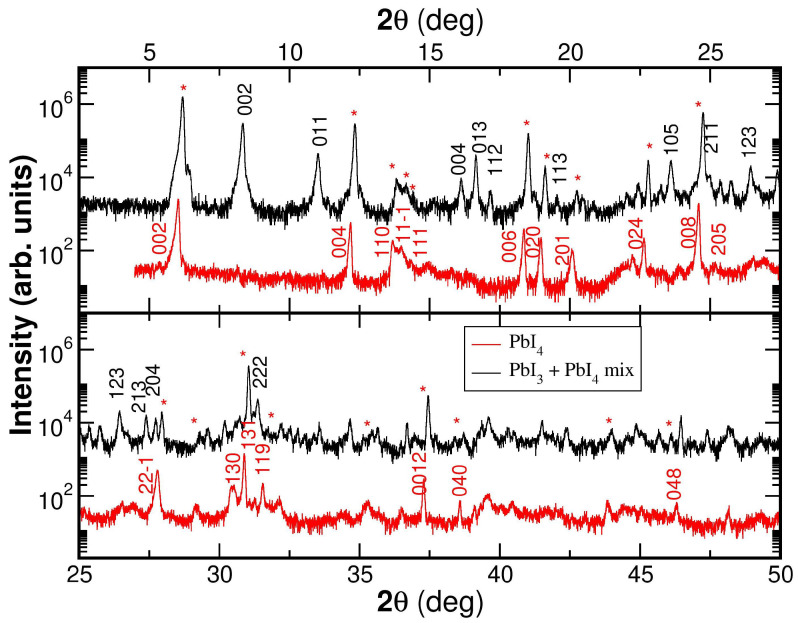
Diffraction pattern of powder samples: in red, growth at room temperature where the pure ${\left(PEA\right)}_{2}{\mathrm{PbI}}_{4}$ phase is formed, and in black, growth at high temperature where a mixture of $\left(PEA\right){\mathrm{PbI}}_{3}$ and ${\left(PEA\right)}_{2}{\mathrm{PbI}}_{4}$ phases is observed. Red star flags point out the ${\left(PEA\right)}_{2}{\mathrm{PbI}}_{4}$ phase Bragg peaks present in the high-temperature powder sample. The X-ray diagram has been divided into two angle ranges for clarity.

We compare the X-ray diffraction patterns of two representative powder samples in Figure 2. The red line corresponds to a sample crystallized at low temperature and related to the 2D-${\left(PEA\right)}_{2}{\mathrm{PbI}}_{4}$ phase. The black line corresponds to a sample crystallized at high temperature and related to the 1D-$\left(PEA\right){\mathrm{PbI}}_{3}$ phase.

In Figure 2, it can be observed that the peaks of the red pattern match perfectly with those described in the literature by Billing et al. (2006) [36] for the 2D-${\left(PEA\right)}_{2}{\mathrm{PbI}}_{4}$, confirming that it is a stable phase at room temperature.

On the other hand, the black diffraction pattern of Figure 2, corresponding to a sample crystallized at high temperature, reflects a greater structural complexity. This indicates the presence of Bragg peaks characteristic of both phases: the 2D-${\left(PEA\right)}_{2}{\mathrm{PbI}}_{4}$ phase and the 1D-$\left(PEA\right){\mathrm{PbI}}_{3}$ phase, confirming a coexistence of both phases. This fact was also described by Billing et al. (2006) [36].

**Figure 3:**
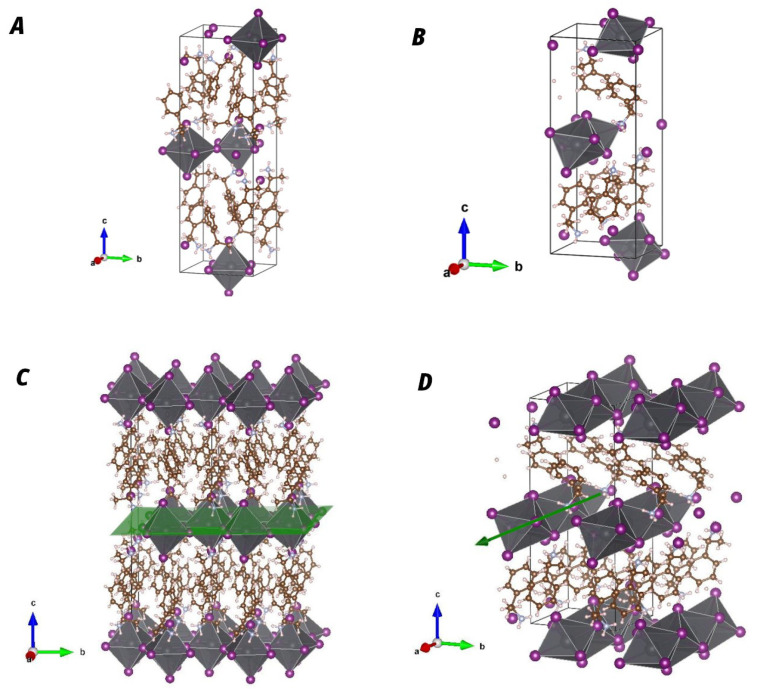
Top panel: Crystal structure unit cell of (**A**) the two-dimensional phase ${\left(PEA\right)}_{2}{\mathrm{PbI}}_{4}$ and (**B**) the one-dimensional $\left(PEA\right){\mathrm{PbI}}_{3}$ phase. Bottom panel: Representation of several unit cells to show in green the dimensionality of the inorganic sublattice: (**C**) the two-dimensional phase ${\left(PEA\right)}_{2}{\mathrm{PbI}}_{4}$; (**D**) the one-dimensional $\left(PEA\right){\mathrm{PbI}}_{3}$ phase.

The crystal structures of both compounds agree with the crystal data recorded at the Cambridge Crystallographic Data Center (CCDC) refs. 607736 and 607737 for the 1D phase and 607740 and 607741 for the 2D phase [36]. The crystal structures of both phases are shown in Figure 3. These lattice units were used for the peak assignment shown in Figure 2. In the lower panel of Figure 3, it is clearly shown with green guides that the ${\left(PEA\right)}_{2}PbI_{4}$ compound has a structure characterized by two-dimensional planes of inorganic octahedra, while the $\left(PEA\right)PbI_{3}$ compound has an arrangement of octahedra in the form of one-dimensional wires. In both structures, the lead–iodide units are organized into octahedra (shown in gray in Figure 3). The difference between the two arrangements is the link between the PbI octahedra.

In the 1D arrangement, the octahedra share faces, meaning that all iodide anions exhibit the same coordination environment. In contrast, in the 2D structure, the octahedra share vertices, allowing us to distinguish between meridional iodide anions (located within the Pb–I plane) and out-of-plane iodide anions (oriented perpendicular to the Pb–I layers), which exhibit lower coordination with Pb cations. As a consequence, the distance between the first-neighbor Pb-Pb changes from 4.05±0.1 Å in the 1D structure to 6.3±0.1 Å in the 2D structure.

The results of X-ray diffraction studies of the synthesized materials indicate a coexistence of both phases ${\left((PEA\right)}_{2}PbI_{4}$ and $\left(PEA\right)PbI_{3})$ depending on the crystallization temperature. This phenomenon indicates that high-temperature growth conditions favor the formation of the $\left(PEA\right){\mathrm{PbI}}_{3}$ phase but do not prevent the residual presence of ${\left(PEA\right)}_{2}{\mathrm{PbI}}_{4}$.

A quantitative analysis of the 2D ${(\mathrm{PEA})}_{2}{\mathrm{PbI}}_{4}$ phase and the 1D ${(\mathrm{PEA})\mathrm{PbI}}_{3}$ phase in the high-temperature powder sample can be performed by comparing the intensity ratio of their characteristic diffraction peaks and the corresponding structure factors. Specifically, the intensity of the peak at $6.15^{{}^{\circ}}$, characteristic of the (002) Bragg reflection of ${\left(\mathrm{PEA}\right)}_{2}{\mathrm{PbI}}_{4}$, is compared with that of the peak at $8.26^{{}^{\circ}}$, characteristic of the (002) Bragg reflection of $(\mathrm{PEA}){\mathrm{PbI}}_{3}$, in order to determine their relative contributions. Since the diffracted intensity is proportional to the square of the structure factor, the measured intensity ratio must be corrected by multiplying it by the square of the ratio of the corresponding structure factors:

(1) $$\frac{I_{{\left(\mathrm{PEA}\right)}_{2}{\mathrm{PbI}}_{4}}}{I_{\left(\mathrm{PEA}\right){\mathrm{PbI}}_{3}}}\cdot \frac{{\left|F_{\left(\mathrm{PEA}\right){\mathrm{PbI}}_{3}}\right|}^{2}}{{\left|F_{{\left(\mathrm{PEA}\right)}_{2}{\mathrm{PbI}}_{4}}\right|}^{2}}.$$

The ratio between the squares of the structure factors is given by

(2) $$\frac{{\left|F_{\left(\mathrm{PEA}\right){\mathrm{PbI}}_{3}}\right|}^{2}}{{\left|F_{{\left(\mathrm{PEA}\right)}_{2}{\mathrm{PbI}}_{4}}\right|}^{2}}=0.816,$$

as calculated using VESTA [43]. Thus, the sample represented in red is identified as pure ${\left(\mathrm{PEA}\right)}_{2}{\mathrm{PbI}}_{4}$, which is stable at room temperature, whereas the sample represented in black, crystallized at high temperature, corresponds to a mixture of both compounds, with ${\left(\mathrm{PEA}\right)}_{2}{\mathrm{PbI}}_{4}$ still accounting for approximately 71% of the powder.

This means that the powder sample crystallized at high temperature following the recipes given by Billing et al. [36] has a maximum of 29 % of the target 1D-$\left(PEA\right){\mathrm{PbI}}_{3}$ phase. Thus, in order to study the influence of the inorganic perovskite part dimensionality on the optoelectronic properties of the perovskite, the powder samples are not valid.

#### 3.1.2. Thin-Film Samples Structure

We sought a procedure to obtain pure and stable 1D-$\left(PEA\right){\mathrm{PbI}}_{3}$ and 2D-${\left(PEA\right)}_{2}{\mathrm{PbI}}_{4}$ for the same sample shape conditions. Thus, we studied the structure by growing thin-film samples. The main difficulty was to obtain a pure high-temperature 1D phase. Two strategies were explored to deposit and synthesize films on a substrate to maximize the 1D-$\left(PEA\right){\mathrm{PbI}}_{3}$ phase: (1) thin-film deposition at high temperature and (2) thin-film growth at room temperature followed by annealing at ${70\;}^{{}^{\circ}}$C for 2 h under a helium atmosphere.

The initial high-temperature sample powder, which we have already shown in Section 3.1.1, contains a mixture of both phases and was dissolved in acetone and deposited on an ITO (Indium Tin Oxide) substrate widely used in electronic devices. Prior to deposition, the ITO substrate was coated with a 30 nm gold layer by evaporation, which improved the wetting behavior of the acetone/perovskite solution.

**Figure 4:**
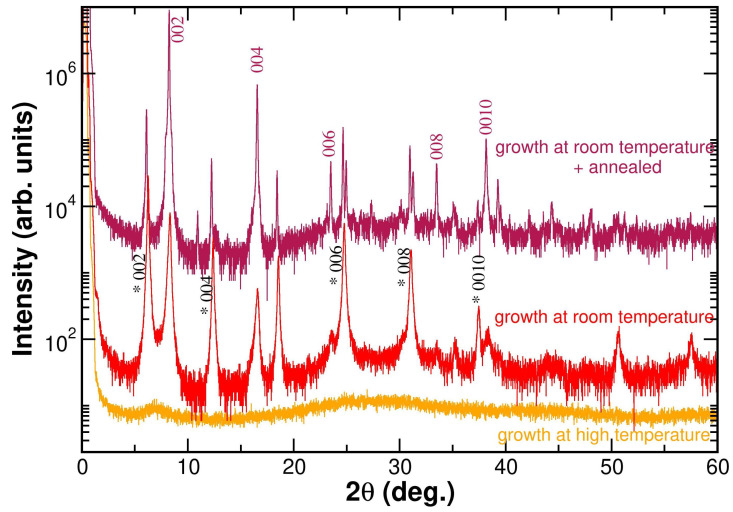
Crystalline structure of the thin films for the 1D structure stable at high temperature: orange line, thin film grown at high temperature, showing an amorphous structure; red line, thin film grown at room temperature without further treatment, showing an identical phase proportion to the initial powder; deep-red line, thin film grown at room temperature and annealed at ${70\;}^{{}^{\circ}}$C for 2 h in a helium atmosphere, showing a significant increase in the 1D-$\left(PEA\right){\mathrm{PbI}}_{3}$ phase. The Bragg peaks of the 2D-${\left(PEA\right)}_{2}{\mathrm{PbI}}_{4}$ phase are marked with black asterisks.

Figure 4 shows the X-ray diffraction patterns of the 2-D phase thin-film samples grown using different thermal annealing procedures (note that this 2D phase is stable at high temperature). The orange-line pattern, which corresponds to the film deposited directly at high temperature, shows a diffraction pattern dominated by a diffuse background signal, indicating an amorphous structure. This result suggests that when the deposition is made on a heated substrate, it does not favor the formation of a well-defined crystalline phase. This behavior is consistent with the idea that high temperatures can promote rapid film growth due to the fast solvent evaporation, preventing the formation of an ordered structure.

The red-line pattern corresponds to the film grown at room temperature. The diffraction pattern shows a mixture of both phases in the same proportion as in the initial powder (see red line in Figure 2). This sample was then annealed at ${70\;}^{{}^{\circ}}$C for 2 h under a helium atmosphere. The resulting sample shows the diffraction pattern shown in the deep-red line of Figure 4. This sample shows a noticeable increase in the intensity of the Bragg peaks associated with the desired 1D $\left(PEA\right){\mathrm{PbI}}_{3}$.

The diffraction pattern obtained for the annealed powder sample exhibits well-defined peaks, representative of a highly ordered crystalline structure. Although traces of the low-temperature phase (2D-${\left(PEA\right)}_{2}{\mathrm{PbI}}_{4}$) are still visible, indicating the presence of some traces of the 2D-phase (note the logarithmic scale of the y-axis of the graph, which enhances its signal). However, the high-temperature 1D-$\left(PEA\right){\mathrm{PbI}}_{3}$ phase clearly predominates.

A quantitative study of the 1D/2D compounds ratio can be made in the annealed thin film. The intensity ratio of the (002) Bragg peaks of both phases in the annealed thin film (located at $6.15^{{}^{\circ}}$ and $8.26^{{}^{\circ}}$ for 2D-${\left(PEA\right)}_{2}{\mathrm{PbI}}_{4}$ and 1D-$\left(PEA\right){\mathrm{PbI}}_{3}$, respectively) shows that only a residual 2.6 % of the 2D ${\left(PEA\right)}_{2}{\mathrm{PbI}}_{4}$ phase remains. This shows that growth at room temperature with subsequent annealing at ${70\;}^{{}^{\circ}}$C for 2 h under a helium atmosphere is effective in synthesizing layers with a predominant 1D crystal structure, effectively suppressing the initial 2D phase.

**Figure 5:**
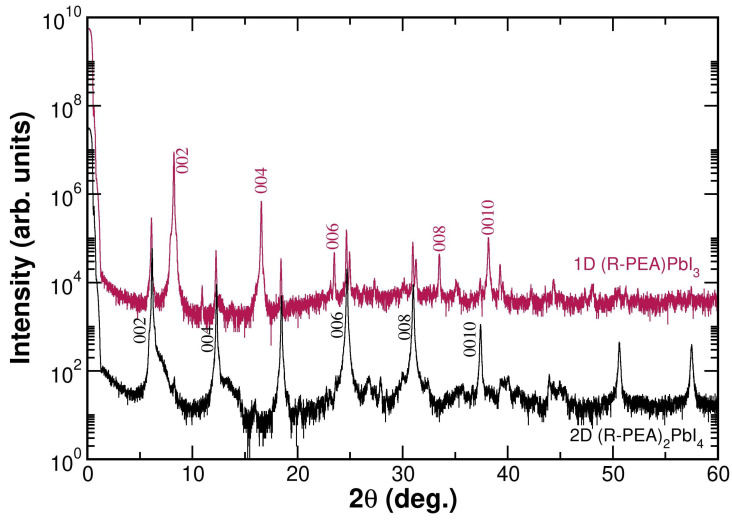
Crystalline structure of thin films of the 2D structure (stable at low temperature, black line) and the thin film of the 1D structure (stable at high temperature after room-temperature growth and annealing, deep-red line).

When the low-temperature powder sample is deposited as a thin film at room temperature under the same conditions, a predominantly 2D ${\left(PEA\right)}_{2}{\mathrm{PbI}}_{4}$ phase is obtained. Figure 5 shows its diffraction pattern (black line) compared to the final 2D phase (red line). This clear distinction in the diffraction patterns highlights the effectiveness of the deposition process in selectively promoting the formation of the desired phase. These thin-film samples are used to compare the influence of the inorganic dimensionality of the perovskite on its final optoelectronic properties.

**Figure 6:**
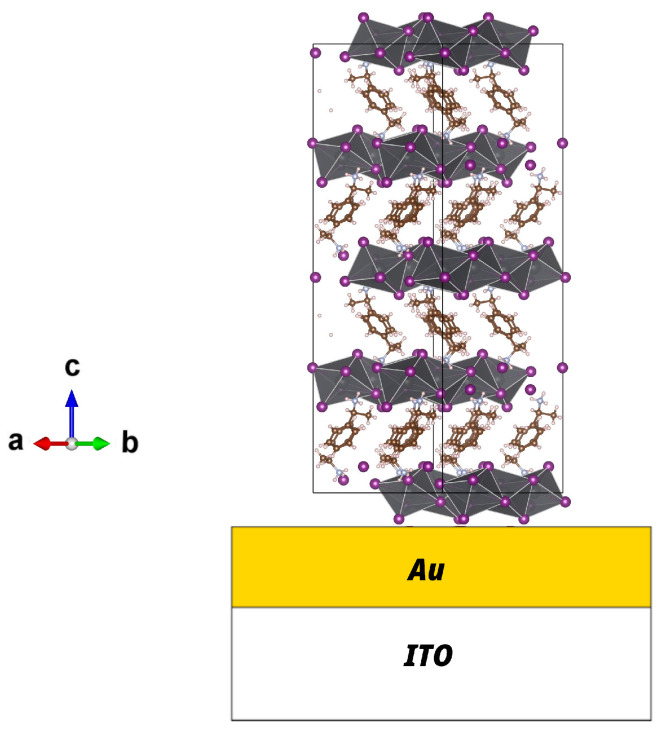
Molecular structure of the $\left(PEA\right){\mathrm{PbI}}_{3}$ thin-film perovskite on a gold-coated ITO substrate surface. In the ${\left(PEA\right)}_{2}{\mathrm{PbI}}_{4}$ thin film, the inorganic planes characteristic of its structure (see Figure 3C) are parallel to the substrate surface.

Both thin-film samples show oscillations at low diffraction angles, indicating a thickness homogeneity of the films (see Figure 5 at 2θ<2). In Figure 5, it can also be observed that only Bragg peaks of the {002} family appear. In the 2D ${\left(PEA\right)}_{2}{\mathrm{PbI}}_{4}$ phase, the presence of Bragg peaks belonging exclusively to this family indicates that the unit-cell *c*-axis is perpendicular to the surface. This implies that the inorganic planes are oriented parallel to the surface (see Figure 3). In the high-temperature sample, only the {002} reflections are also observed in the θ/2θ pattern, indicating that its crystallographic *c*-axis is also oriented perpendicular to the surface. Therefore, the 1D-$\left(PEA\right){\mathrm{PbI}}_{3}$ inorganic conductive wires are aligned parallel to the substrate surface (see Figure 6).

The crystalline quality along the direction perpendicular to the surface can be estimated using the Scherrer equation [44]. From the full width at half maximum (FWHM) of the first diffraction peak for the $\left(PEA\right){\mathrm{PbI}}_{3}$ thin film (being $\Delta \theta =0.0792\pm 0.0012^{{}^{\circ}}$ for the peak located at $\theta =4.126\pm 0.001^{{}^{\circ}}$), we calculate a domain size in the z-direction of 1050.4±0.3 Å. Applying this crystallographic analysis to the ${\left(PEA\right)}_{2}{\mathrm{PbI}}_{4}$ thin film (which exhibits a first diffraction peak at an angular position of $\theta =3.08\pm 0.001^{{}^{\circ}}$ and a FWHM of $\Delta \theta =0.079\pm 0.0012^{{}^{\circ}}$) gives a domain size of 1053.2±0.3 Å, which is very similar to that obtained for the $\left(PEA\right){\mathrm{PbI}}_{3}$ film. These values are comparable to those extracted from the diffraction analysis of the low-temperature powder sample (${\left(PEA\right)}_{2}{\mathrm{PbI}}_{4}$). In this case, the first diffraction peak shows an angular width (FWHM) of $\Delta \theta =0.086\pm 0.009^{{}^{\circ}}$, resulting in a domain size of 966.2±0.3 Å.

The fact that both the 1D and 2D thin-film phases exhibit the same out-of-plane orientation is crucial because it allows a direct comparison of how the inorganic dimensionality of the HOIP influences the photoresponse of the perovskite.

### 3.2. The HOIP Photoresponse: SPV Effect

Once the synthesis conditions for both 1D and 2D PEA perovskites in powder and thin film form have been established, we can compare how the photocurrent depends on the dimensionality of the inorganic framework ordering. This analysis would enable us to assess how, for nearly identical organic and inorganic chemistries, the electronic behavior and the photoresponse of the perovskite are governed by its crystal structure dimensionality, specifically comparing $2D-{\left(PEA\right)}_{2}{\mathrm{PbI}}_{4}$ and $1D-\left(PEA\right){\mathrm{PbI}}_{3}$. Furthermore, we propose that elucidating how the electronic properties and photoresponse vary with dimensionality may provide valuable insights into the mechanisms governing charge transport in these hybrid organic–inorganic materials.

**Figure 7:**
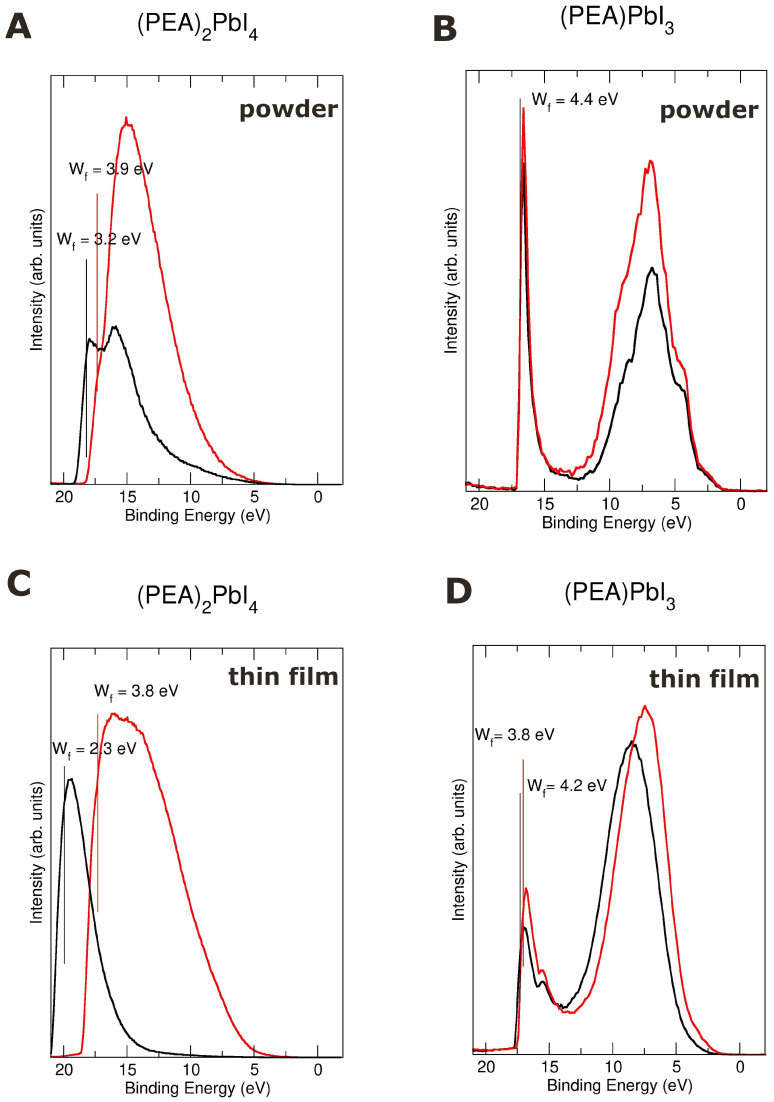
Dependence of the UPS HeI-SECO spectra of 1D and 2D PEA perovskites (**left** and **right** panels, respectively) in powder and thin-film samples (**upper** and **bottom** panels, respectively) with an external illumination visible light source. Red lines when the light is on and in black when it is off.

To thoroughly investigate the photocurrent mechanisms responsible for the pronounced photoresponse observed in these HOIP [16], we performed a series of experimental measurements on both structures under varying illumination conditions. Figure 7 shows ultraviolet light-induced photoemission spectroscopy (UPS) measured with and without the stimulation of an external visible light source.

In this context, it is important to note that the absorption spectrum of ${\left(\mathrm{PEA}\right)}_{2}{\mathrm{PbI}}_{4}$ has been previously calculated and measured [45,46]. The absorption spectrum exhibits a peak at 2.3 eV, corresponding to a wavelength of λ=538.65 nm, which lies in the green region of the visible spectrum. We investigated the wavelength dependence of the light-induced UPS response of ${\left(\mathrm{PEA}\right)}_{2}{\mathrm{PbI}}_{4}$ using an RGB laser source. Under red-light illumination ($\lambda_{\mathrm{red}}=635\;\mathrm{nm}$), no observable effect is detected. Under green-light illumination ($\lambda_{\mathrm{green}}=520\;\mathrm{nm}$), the effect is partially observed, whereas under blue-light illumination ($\lambda_{\mathrm{blue}}=450\;\mathrm{nm}$) the effect is fully developed and identical to that observed under “white” light illumination. Therefore, we conclude that the light on–off transient experiments can be reliably performed using the white LED source for sample illumination.

During experiments performed under sample illumination, no changes in sample temperature, base pressure, or data reproducibility were observed that would suggest light-induced sample degradation.

Figure 7 shows a clear shift of the SECO edge position toward lower binding energies when the illumination is switched off in the ${\left(\mathrm{PEA}\right)}_{2}{\mathrm{PbI}}_{4}$ samples, with a more pronounced shift observed in the thin-film sample. This increase in the work function is characteristic of a p-type deflection of the electronic bands SPV. That suggests surface band bending compensated for by the generation of photoinduced charge. A similar behavior has already been reported in other 2D lead–halide compounds formed by stacking of PbI-planes [16,47]. Lead–halide-based perovskites show “anomalies” in light absorption processes. In PbI_2_, which also exhibits such anomalies, it was demonstrated that a detailed study of this behavior can provide valuable information about the mechanism governing their photoresponse [47]. The authors concluded that in the PbI_2_ case, these processes can be understood as a surface photo-voltage effect created by the appearance of a positive electric field near the surface, originating from positively charged vacancies due to halide sublimation. The analysis of the variation in the photoemission signal during light on–off transients provides information on the mobility of both majority and minority charge carriers within the semiconductor [16,47].

The XPS results are consistent with these observations; however, because XPS probes deeper electronic levels rather than those near the Fermi level, as in UPS, being these closer to the Fermi level the most implicated in the photoresponse with visible light, we do not show them here (see Appendix A).

In the 1D-$(\mathrm{PEA}){\mathrm{PbI}}_{3}$ samples, the situation is different: the work function is less affected by the presence or absence of external light stimuli, being almost negligible in the powder sample. In the 1D-$(\mathrm{PEA}){\mathrm{PbI}}_{3}$ thin-film sample, a shift of 0.4 eV in the work function is observed; however, a larger shift is observed in the UPS peak placed at ≈7 eV (see Figure 7D), indicating that the band bending is not linear for all electron states. In all studied samples (1D-$(\mathrm{PEA}){\mathrm{PbI}}_{3}$ and 2D-${\left(\mathrm{PEA}\right)}_{2}{\mathrm{PbI}}_{4}$ phases in both powder and thin film form), an increase in the peak intensities under external light stimuli occurs.

**Figure 8:**
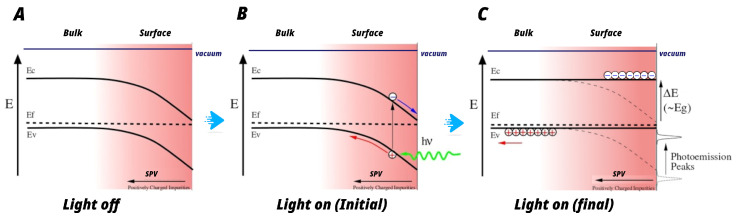
Sketch of the process of electron and hole excitation and relaxation when the light is on and off. The diagram shows the surface photo-voltage effect in a p-type semiconductor in the absence of light (**A**); at the initial steps after illumination (**B**); and once reached the saturation after the illumination moment (**C**) in a p-type semiconductor. The first step of the process, when the external visible light source is turned on (**B**), is shown for comprehension.

Considering these results, we propose a photoactivation mechanism that explains the observed phenomena under external light stimulation (Figure 8). This electronic behavior is similar to that of other organic lead–halide perovskites reported in the literature [16,48]. As we have already pointed out, all the observed shifts of the electronic structure with light (shift of core and valence band peaks towards lower binding energy under illumination) indicate a p-type deflection of the majority carriers for the XBA perovskites studied. Additionally, the work function increases when the samples are illuminated. These facts indicate that the PEA-perovskite samples have band bending at the surface. This band bending is due to the presence of a surface photo-voltage (SPV) effect. Under illumination, the electrons are promoted to the conduction band, resulting in flat bands.

Considering the wavelength range of the external light source used in the experiment (visible light from 430 nm to 700 nm), which energetically covers the theoretical band gap of 2D-${\left(\mathrm{PEA}\right)}_{2}{\mathrm{PbI}}_{4}$ ($E_{g}=2.08\;\mathrm{eV}$ [45]), and the measured work-function shift of 1.5 eV for the thin-film sample, it can be inferred that the Fermi level of the perovskite is located at approximately 72% of the ${\left(\mathrm{PEA}\right)}_{2}{\mathrm{PbI}}_{4}$ band gap. This result therefore suggests that the Fermi level lies relatively close to the conduction band, indicating a p-type surface photo-voltage (SPV) effect.

The lower work-function shift observed in the 1D-$(\mathrm{PEA}){\mathrm{PbI}}_{3}$ perovskite implies a lower SPV effect compared to the 2D counterpart. This phenomenon can be explained by the greater stability of iodine atoms within the one-dimensional structure. In this configuration, the iodine atoms exhibit a higher coordination than in the 2D structure, as discussed in Section 3.1.1. A higher coordination of the iodine atoms reduces the likelihood of iodine vacancy formation. Since the electric field responsible for the SPV effect comes from the presence of iodine vacancies, an increased chemical stability of iodine results in a reduced SPV effect. Although this effect may originate from other mechanisms, previous experiments performed on ${\mathrm{PbI}}_{2}$ and other 2D-HOIPs [16,47] indicate that this explanation can be considered the most plausible scenario for the observed lower SPV. Consequently, the reduced stability of these vacancies in the 1D perovskite leads to diminished charge-carrier concentration, lifetime, and mobility. This increases the stability of the compound under light stimulation but reduces the effectiveness of the perovskite for photocurrent generation. New strategies aimed at enhancing anion vacancy stability must therefore be explored.

Figure 8 illustrates the photoactivation mechanism in perovskites under illumination, highlighting three key processes:

- With light off, positively charged iodide vacancies $\left(I^{-}\right)$ in the near-surface region generate an electric field that points from the surface to the volume of the material, resulting in the appearance of an electrical potential that causes band bending. These defects favor the location of holes and generate an electron level within the band gap associated with these vacancies. This charge distribution generates a characteristic p-type SPV (see left panel of Figure 8). This depends on the stability of the anion vacancies promoted by the PbI-octahedron sharing present in their respective structures.
- When the material is illuminated, photons generate electron–hole pairs (excitons). The electric field at the surface separates these charges: electrons migrate to the surface, neutralizing positively charged vacancies, and holes diffuse into the bulk. The accumulation of electrons on the surface reduces band bending, driving the system toward a flat-band condition (see right panel of Figure 8). This manifests as a shift of the electronic levels toward lower binding energies.
- When the light is turned off, the previously generated electron–hole pairs tend to recombine. This recombination does not occur directly, but through intermediate energy levels located within the gap, which are associated with iodide vacancies $\left(I^{-}\right)$.

**Figure 9:**
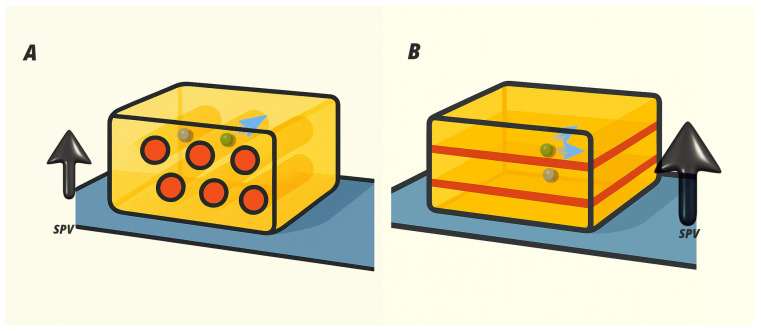
Sketch of the inorganic wires (**A**) and planes (**B**) of the 1D and 2D PEA-perovskite, respectively, with respect to the substrate surface. The SPV direction points perpendicular to the substrate surface. In the figure, we represent the hole (gray) and electron (green) diffusion within the perovskite sample.

The photoactivity mechanism involves a three-step process: first, the excitation of charge carriers leads to a transient increase in conductivity due to the generation of electron–hole pairs; second, the migration of these carriers; and third, their recombination, which can be affected by the SPV momentum. To better understand the dynamics of generation, diffusion, and recombination of carriers in the perovskite, we compare the behavior of the powder sample with the thin film because here the structural dimensionality and SPV momentum have a well-defined orientation. Figure 9 shows the relative directions of the wires/planes and the SPV for the $(\mathrm{PEA}){\mathrm{PbI}}_{3}$ and ${\left(\mathrm{PEA}\right)}_{2}{\mathrm{PbI}}_{4}$ thin films. This defined orientation of the inorganic diffusion wires/planes and the SPV is crucial for analyzing the role of the diffusion dimensionality and the SPV direction in the light photoresponse relaxation phenomenon. In any case, additional factors must be considered to explain the different effects observed in the 1D and 2D PEA lead–halide perovskites.

#### Photoresponse: Kinetics and Transient Adjustment

**Figure 10:**
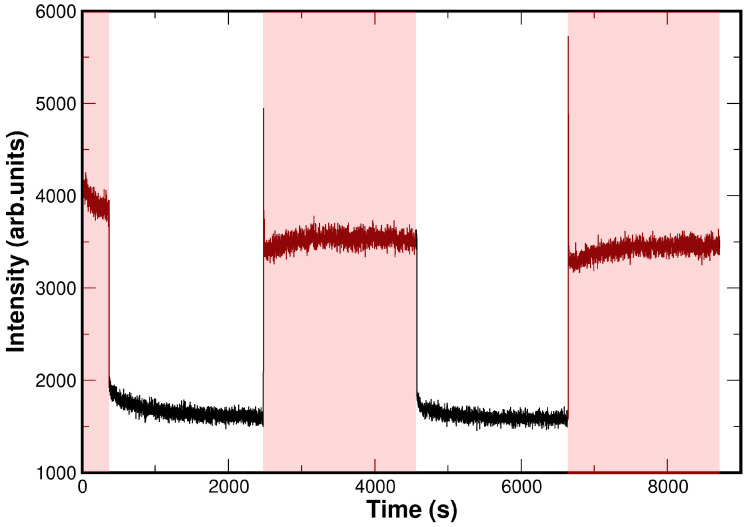
The reversible character of the photocurrent phenomenon produced under external light illumination is followed in the HeI-SECO spectra for the 2D-${\left(PEA\right)}_{2}{\mathrm{PbI}}_{4}$ powder sample used as an example. Several cycles of light on/off are shown. The red shaded areas indicate when the external illuminating light is turned on. In the figure, the transient “anomalies” measured at the edges when the external light is turned on can be seen.

**Figure 11:**
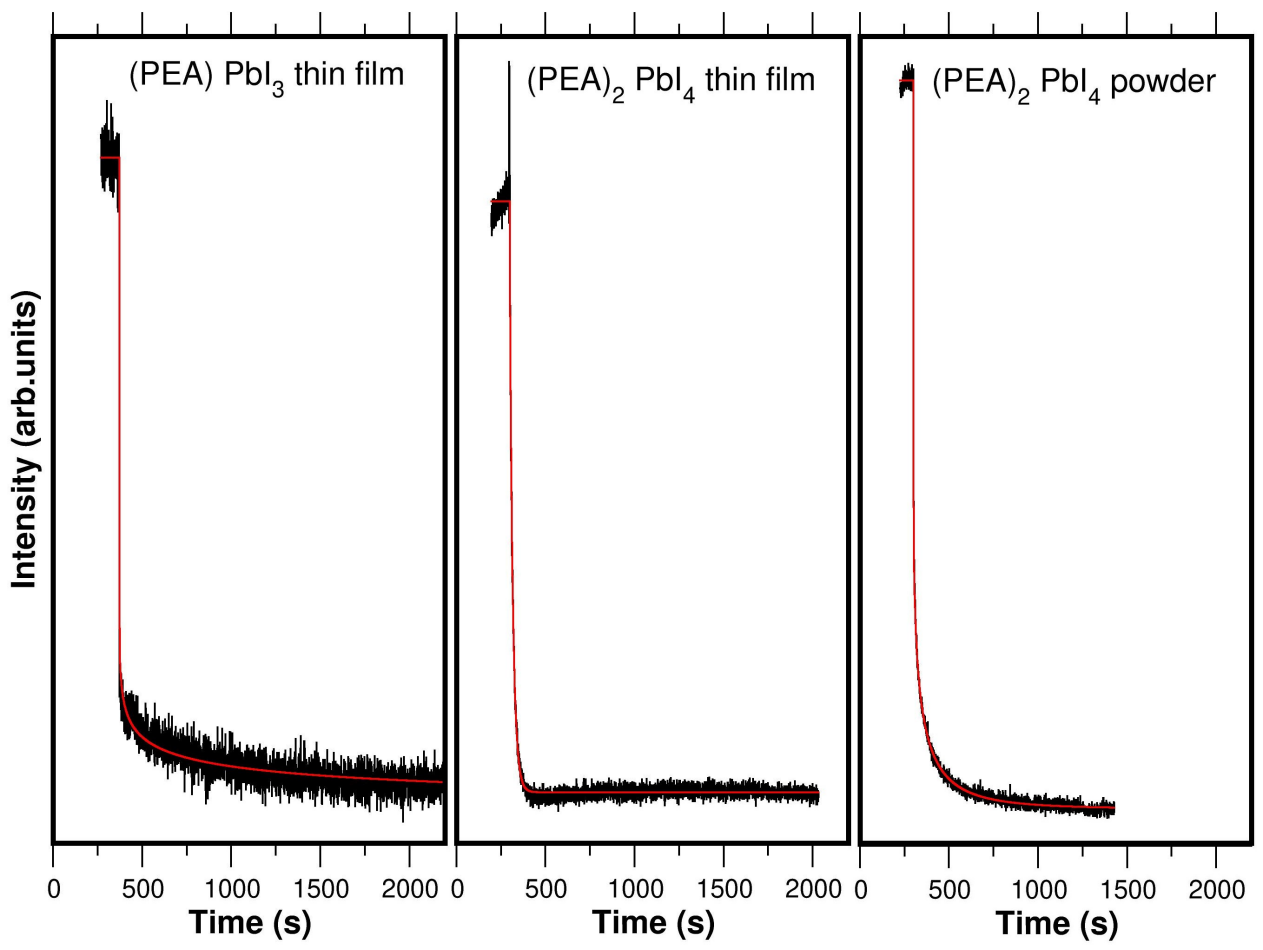
Transients of HeI-SECO intensity in $(\mathrm{PEA}){\mathrm{PbI}}_{3}$ and ${\left(\mathrm{PEA}\right)}_{2}{\mathrm{PbI}}_{4}$ thin layers when external light is turned off. We use the ${\left(\mathrm{PEA}\right)}_{2}{\mathrm{PbI}}_{4}$ powder sample as reference to see if sample reorientation affects the relaxation process. The experimental data are shown in black and the fit in red.

**Figure 12:**
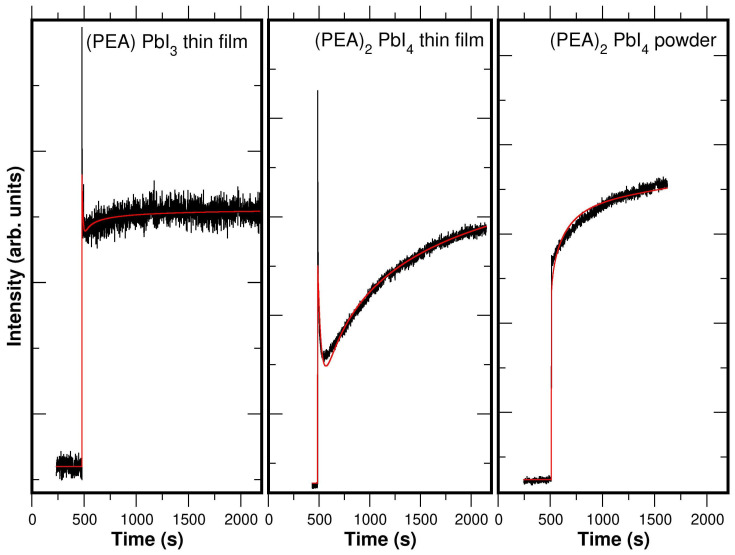
Transients of HeI-SECO intensity in $(\mathrm{PEA}){\mathrm{PbI}}_{3}$ and ${\left(\mathrm{PEA}\right)}_{2}{\mathrm{PbI}}_{4}$ thin layers when external light is turned on. We use the ${\left(\mathrm{PEA}\right)}_{2}{\mathrm{PbI}}_{4}$ powder sample as reference to see if sample reorientation affects the relaxation process. The experimental data are shown in black and the fit in red.

Comparing the temporal evolution of the perovskite photoresponse in the 1D and 2D perovskites can provide insight into the limiting process governing the photoresponse relaxation. The decay is extremely slow (on the order of several minutes), allowing it to be monitored using photospectroscopic techniques, which are not ultrafast methods. As we have already shown in Figure 7, the UPS of the PEA lead–halide perovskites changes in position and intensity under light illumination. Thus, monitoring the UPS intensity at a fixed energy position allows tracking the temporal evolution of photon induced states and the associated relaxation dynamics [47]. It has been verified that the process is completely reversible with cycles of light on/off (see Figure 10). The aim is to determine the influence of the inorganic dimensionality on the photoresponse decay kinetics (see the experimental data in Figure 11 and Figure 12).

This dynamic can be modeled using diffusion equations for both types of carriers, allowing parameters such as relaxation time (τ) to be extracted. Various models were used to fit the experimental kinetic data, from simple to more complex exponential equations.

The best fit when the light is turned off is obtained with a stretched-exponential decay function, known as the Kohlrausch–Williams–Watts stretched function [49]:

(3) $$V\left(t\right)=V_{1}\;e^{-{\left(\frac{t}{\tau_{1}}\right)}^{\beta }}+V_{2}\;e^{-\left(\frac{t}{\tau_{2}}\right)}$$

where (0<β<1). The first term represents the stretched-exponential decay. Compared to a simple exponential decay (where β=1), this decay is initially faster but takes longer to reach its asymptotic value. Therefore, the overall shape of the curve decay changes depending on the stretching value β. Kohlrausch et al. [49] used this stretched-exponential function to explain the decay of the residual charge in a glass Leyden jar in experiments related to the relaxation of complex electronic and molecular systems, and more recently, it has proven to be an effective tool to describe this super-slow perovskite photoactivity process [16,50,51] determined by its structural dimensionality. In general, this function is applicable when a process deviates from simple first-order kinetics, where the decay rate is not constant but decreases with time as $(t^{-\beta }$) [52,53]. The stretched-exponential decay is also observed in the electronic transport of other materials such as semiconductors, nanocrystalline materials, and conjugated polymers due to molecular/crystal relaxation, trap states, or disorder [49]. In general, β is associated with dimensional confinement, although it also reflects disorder and trap-distribution effects.

The equivalent stretched-exponential equation when the light is on is given by:

(4) $$V\left(t\right)=V_{1}\left(1-e^{-{\left(\frac{t}{\tau_{1}}\right)}^{\beta }}\right)+V_{2}\;e^{-\frac{t}{\tau_{2}}}$$

The photoconductive description based on the stretched-exponential model is not the only possible approach. In particular, models based on fractional photoconductivity [54] may offer an interesting alternative framework. However, in the present work, we choose to retain the stretched-exponential model due to the direct relationship between the β parameter and the transport dimensionality already shown in the literature [16,47,50,51]. A detailed analysis of the experimental data within the framework of fractional photoconductivity models will be addressed in future studies.

The resulting fitted curves are shown in Figure 11 and Figure 12, and the corresponding parameter values are given in Table 1. A significant observation is that the stretched-exponential decay parameter (β) is equal to 0.5±0.05 for all 2D-${\left(\mathrm{PEA}\right)}_{2}{\mathrm{PbI}}_{4}$ samples, while β=0.15±0.01 for the 1D-$(\mathrm{PEA}){\mathrm{PbI}}_{3}$ samples, for both light-on and light-off conditions. This agrees with the literature [50,51], which reports β=1 for 3D perovskites, highlighting the correlation between β and the structural dimensionality of the charge-transport channel. These results agree with the conclusion that for 2D perovskites (β≈0.5), the transport is essentially two-dimensional. This finding aligns with the PbI_2_ system, where transport is defined by the existence of 2D PbI-planes [47]. The PbI-octahedron planes act as 2D reservoirs for charge carriers, restricting conduction. Moreover, this implies the presence of various trap states at plane interfaces or powder-grain surfaces, where electron trapping is linked to 2D transport among trap states [55].

Supporting this interpretation, the β≈0.15 value for $(\mathrm{PEA}){\mathrm{PbI}}_{3}$ suggests a one-dimensional transport mechanism for the 1D perovskite. This results in a flatter relaxation kinetics in the 1D perovskite compared to the ${\left(\mathrm{PEA}\right)}_{2}{\mathrm{PbI}}_{4}$. Thus, this suggests that the inorganic structural dimensionality has a key role in the photoresponse relaxation. This behavior could be explained by the stronger quantum confinement effect, as reducing the structural dimensionality, from 2D to 1D, leads to faster charge carrier [56]. The lower dimensionality of the $(\mathrm{PEA}){\mathrm{PbI}}_{4}$ (1D) structure reduces electron mobility by increasing the energetic barrier for charge transport, leading to a slower relaxation dynamic compared to the ${\left(\mathrm{PEA}\right)}_{2}{\mathrm{PbI}}_{4}$ thin film (2D). Thus, the limiting process of the relaxation dynamic is the carrier diffusion rather than the electron–hole recombination.

According to theoretical SPV models, the parameter $\tau_{1}$ is related to the rate of charge diffusion. In Table 1, it can also be observed that the higher values for $\tau_{1}$ and $\tau_{2}$ parameters occur when light is off for all studied samples. Therefore, it can be concluded that at short times in the light-on transient, the system is dominated by an auto-decelerated electron diffusion term, while the light-off transient can be modeled by a diffusion-type term for holes. In both long-term models, the kinetics are dominated by the relaxation time of the impurity levels located at the perovskite bandgap. However, it should be noted that $\tau_{1}$ and $\tau_{2}$ parameters have larger error in the fit, in opposition to the β, which is very well-defined, making it difficult to make further interpretations. Nevertheless, their trends can be analyzed.

The $\tau_{1}$ parameter is likely associated with the mobility of the minority carriers when the light is on, i.e., the electrons, and the mobility of the majority carriers when the light is off, i.e., the holes [47]. In Table 1, it can be observed that $\tau_{1}$ is roughly 6–10 times bigger under light conditions in all studied samples. That means a bigger electron mobility. At the same time, in the 2D samples, the charge carriers have greater mobility. It should be emphasized that in the present context, the term “mobility” does not refer to the carrier mobility (µ), but rather to the charge-transport “capability” of majority and minority carriers determined by their relaxation time.

The $\tau_{2}$ parameter is associated with the electron–hole recombination process. In Table 1, it is shown that this process is more efficient when light is turned on. Considering the relative orientation of the conduction planes and wires with respect to the SPV direction in the thin films, SPV magnitude (see Section 3.2) should affect the recombination process.

In addition, normalized chi-square values are adequate in most cases, except for ${\left(PEA\right)}_{2}PbI_{4}$ thin film, where the fit does not fully reproduce the transient peak (it is very abrupt), although it is phenomenologically correct. This suggests that while the model described by Equations (3) and (4) fits the experiments satisfactorily, it may require further refinement to capture all the physical aspects involved.

By fitting the photoresponse transients measured under light-on and light-off conditions, we obtain key kinetic parameters that are indicative of charge transport in the perovskite, such as the exponent β, which is associated with the dimensionality of the transport in the perovskite, the $\tau_{1}$ parameter, which is associated with the charge diffusion length of the carriers through the diffusion coefficient. The diffusion coefficient of charge carriers is defined as a function of mobility, Boltzmann’s constant, temperature, and electron charge, using Einstein’s relation:

(5) $$D=\frac{\mu k_{B}T}{e}$$

Using this Equation and considering that these compounds have ambipolar character in such a way that electrons and holes have the same mobility [57]. Thus, we take an average value of electron and hole mobility in these compounds ($\mu =1{\mathrm{cm}}^{2}V^{-1}s^{-1}$ by Milot et al. [58]), obtaining an average diffusion coefficient of $D=0.025851\;{\mathrm{cm}}^{2}s^{-1}$. However, it is important to note that, due to the absence of reported mobility values for the 1D perovskite, we have used the diffusion coefficient from the previous one, which is reported for the 2D perovskite. Therefore, the diffusion coefficient values for the 1D system calculated using the preceding equation should be considered an estimate.

From this *D* value and knowing the relaxation times of electrons and holes ($\tau_{1}$ with light on and off, respectively), we can obtain the diffusion length (LD), which is the average distance that a charge-carrier travels in a material before recombining [59]:

(6) $$LD=\sqrt{zD\tau }$$

where z=2,4, or 6 depending on the conduction dimensionality (1D, 2D, or 3D, respectively).

Thus, we can estimate the diffusion length of the charge carriers: electrons in the transients of the light on and holes in the transients of the light off. The $\tau_{1}$ parameter is associated with charge diffusion and is used to calculate the diffusion length of the carriers. The calculated values are shown in Table 2. These diffusion lengths represent upper bounds, as the mobility value is taken from the literature and may differ in our PEA-based samples.

In Table 2, it can be observed that the LD values are significantly higher under light illumination (electrons) than in its absence (holes). This indicates that electron and hole mobility lengths are different in these materials despite their reported ambipolar character [57]. Regarding diffusion lengths, comparable values are obtained for all the studied samples, being slightly lower in the $(\mathrm{PEA}){\mathrm{PbI}}_{3}$ thin-film sample, which shows a lower LD.

The lower diffusion length in the 1D perovskite compared to the 2D counterpart is consistent with the difference in diffusion observed between 3D and 2D perovskites shown in the literature [58], as well as the lower SPV in this case. However, no significant differences were found between the 2D powder and thin-film samples despite the increased directionality of the confinement axis relative to the SPV direction.

Our findings are consistent with, and therefore reinforce, the results presented in a recent review in the field [60], where the authors highlight the potential of perovskite thin films for optoelectronic applications. The authors suggest that the ${\mathrm{MAPbI}}_{3}$ crystalline thin films are promising for high-speed and broadband photodetectors through optimization of carrier collection at interfaces, whereas quasi-2D perovskite thin films are suitable for high luminance LEDs due to improved carrier confinement and injection. The review further suggests that the development of new bifunctional organic cations could enhance the performance of quasi-2D perovskite-based LEDs.

## 4. Conclusions

In this work, we demonstrate that the dimensionality of the inorganic lead–iodide framework could play a role in the optoelectronic response of hybrid organic–inorganic perovskites. By controlling the crystallization and deposition conditions, we obtained nearly phase pure 1D-$\left(PEA\right)PbI_{3}$ and 2D-${\left(PEA\right)}_{2}PbI_{4}$ phases in both powder and thin film form, allowing us to disentangle the influence of dimensional confinement on their electronic behavior. We have identified a method to grow and stabilize thin films of $\left(PEA\right)PbI_{3}$ on metallic substrates. These films have a structural quality comparable to the thin films obtained for the stable low-temperature phase. This development could enable the use of these one-dimensional compounds in technological applications.

UPS measurements reveal that both compounds exhibit a surface photo-voltage under visible illumination, but with markedly different amplitudes: the 2D perovskite displays a much larger work function shift than its 1D counterpart. In the 2D perovskite, a pronounced photoinduced work-function shift is observed, consistent with significant band bending near the Fermi level. These differences could be related to the distinct coordination environments of iodide ions in each lattice, which influence their stability and, consequently, the density of iodine vacancies responsible for the SPV effect. In the 1D structure, the lower tendency to the formation of iodide defects could be related to structural stability, but also limits the extent of photoinduced surface charge separation.

Time resolved measurements show that the relaxation kinetics follow a stretched exponential behavior, with characteristic exponents associated with inorganic dimensionality (β≈0.5 for 2D, β≈0.15 for 1D). These parameters indicate distinct charge transport regimes, ranging from quasi-two-dimensional diffusion in ${\left(PEA\right)}_{2}PbI_{4}$ to a strongly confined one dimensional transport in $\left(PEA\right)PbI_{3}$. Diffusion lengths extracted from the kinetic fits are consistent with these trends. This suggests that the rate-limiting process for the perovskite relaxation after light illumination is the diffusion of charge carriers. The stretched-exponential model proposed here can describe all major aspects of the photoresponse kinetics.

Altogether, these results suggest a relationship between structure and electronic properties, linking dimensional confinement, defect stability, charge transport, and SPV behavior. This insight offers a potential basis for the rational design of low dimensional perovskites tailored for specific optoelectronic functionalities, especially in architectures where defect robustness or strong directional charge transport are required.

These results highlight the need to balance the dimensionality-induced enhancement of photoelectronic properties with the stability of the ionic defects responsible for the SPV. The 1D perovskite exhibits a lower photoinduced response, which we suggest may be related to a reduced tendency for iodide-vacancy formation. This reduced defect formation can enhance structural stability, suggesting that low dimensional perovskites could find potential applications in devices where long term defect stability is required, such as specific diode or rectifying architectures.

## Figures and Tables

**Table 1:** Parameters obtained from the adjustment of the SPV transients using the Kohlrausch–Williams–Watts [49] function for the different samples under light off and on conditions. Included are the relaxation times $\tau_{1}$ and $\tau_{2}$, the exponent β, the adjustment coefficient $\chi^{2}$, calculated from Equations (3) (light off) and (4) (light on).

Transient	Material	$\chi^{2}$	$\tau_{1}$	β	$\tau_{2}$
	**(PEA)**		**(s)**		**(s)**
	${\mathrm{PbI}}_{3}$ thin film	1.15	2.8±0.3	0.14±0.01	0.5±0.1
light off ($h^{+}$)	${\mathrm{PbI}}_{4}$ thin film	1.69	10.8±1.5	0.56±0.05	1.8±0.3
	${\mathrm{PbI}}_{4}$ powder	1.26	6.8±0.8	0.48±0.05	0.9±0.1
	${\mathrm{PbI}}_{3}$ thin film	1.50	29.7±1.0	0.17±0.03	15±3
light on ($e^{-}$)	${\mathrm{PbI}}_{4}$ thin film	2.57	690.0±80	0.43±0.09	56±7
	${\mathrm{PbI}}_{4}$ powder	2.15	70.8±4.0	0.42±0.12	36±2

**Table 2:** Diffusion Length obtained from the $\tau_{1}$ values obtained from the fit of the photoresponse transient using Equation (6). The LD error was calculated using the equations given in Appendix B.

	Material (PEA)	LD (mm)
	${\mathrm{PbI}}_{3}$ thin film	3.8±0.3
Transient light off ($h^{+}$)	${\mathrm{PbI}}_{4}$ thin film	10.5±1.2
	${\mathrm{PbI}}_{4}$ powder	8.4±0.9
	${\mathrm{PbI}}_{3}$ thin film	12.4±2.3
Transient light on ($e^{-}$)	${\mathrm{PbI}}_{4}$ thin film	84.5±8.1
	${\mathrm{PbI}}_{4}$ powder	27.0±2.1

## Data Availability

The article will be available at https://digital.csic.es/ and https://repositorio.uam.es (accessed on 29 April 2026). All the data supporting this article should be available at https://edatos.consorciomadrono.es/dataverse/Madrono (accessed on 29 April 2026) with https://doi.org/10.21950/MG6HAH.

## Abbreviations

The following abbreviations are used in this manuscript:

UHV	Ultra-High Vacuum
XPS	X-ray Photo Spectroscopy
UPS	Ultraviolet Photon Spectroscopy
SPV	Surface Photo-Voltage
HOIP	Hybrid Organic–Inorganic Perovskite
PSCs	Perovskite Solar Cells
PEA	R-1-PhenylBenzylAmine

## Appendix A. XPS

We also performed X-ray photoemission spectroscopy (XPS) measured under ultra-high vacuum (UHV) conditions. The vacuum chamber is equipped with an X-ray source with a Mg anode whose $K_{\alpha }$ emission line produces photons of energy hν = 1253.6 eV that are used for those measurements. The vacuum chamber is provided witha hemispherical energy analyzer (LEYBOLD LHS10) used for the photoemission experiments. The pass energy of the analyzer was set to 50 eV for the XPS measurements to reach a resolution of 0.7 eV. All the core levels are referred to the $\mathrm{Ag}3d_{5/2}$ core level of the Ag (100) single crystal.

We observe also a photoresponse under external light illumination that agrees with the UPS results. However, as in the XPS, the electronic levels implicated are deep, and the photo response is more evident in the UPS. This is due to the fact that in UPS, the electronic levels are closer to the Fermi level. In this phenomenon, the valence and conduction bands are the most implicated in the photo excitation processes [16,47].

## Appendix B. LD Error Calculation

The error associated with the diffusion length, the value of which is obtained with equation (LD)(3), is determined by error propagation from the uncertainties in D and $\tau_{1}$ (ΔD and $\Delta \tau_{1}$, respectively);

$$\Delta LD=\frac{1}{2}LD\sqrt{{\left(\frac{\Delta D}{D}\right)}^{2}+{\left(\frac{\Delta \tau_{1}}{\tau_{1}}\right)}^{2}}$$
